# Autoimmune Polyendocrinopathy in a Pediatric Patient Presenting With Multisystem Inflammatory Syndrome in Children (MIS-C)

**DOI:** 10.7759/cureus.38407

**Published:** 2023-05-01

**Authors:** Chaitanya Sambangi, Patrice Collins, Julisa Patel, Jacqueline Chan

**Affiliations:** 1 Pediatrics, Medical College of Georgia at Augusta University, Augusta, USA; 2 Pediatric Rheumatology, Children's Hospital of Georgia at Augusta University, Augusta, USA; 3 Pediatric Endocrinology, Children's Hospital of Georgia at Augusta University, Augusta, USA

**Keywords:** graves' disease, pediatric case, covid-19, endocrine disorders, autoimmune polyendocrine syndrome ii, addison's disease, type i diabetes mellitus, aps type ii, multi-system inflammatory syndrome in children (mis-c)

## Abstract

Multisystem inflammatory syndrome (MIS) is a well-known potential sequela of COVID-19 infection. Though prevalence is higher in certain populations, this syndrome is a rare occurrence in children. Beyond MIS, there has been increasing research into COVID infection and the subsequent onset of autoimmune conditions, such as diabetes. However, evidence of a poly-endocrinopathy developing after COVID infection is lacking, and evidence within the pediatric population is virtually nonexistent. In this case, we present the evolution of an autoimmune polyglandular syndrome (APS) type 2 phenotype, consisting of type 1 diabetes, Graves' disease, and adrenal insufficiency, after diagnosis of multisystem inflammatory syndrome of children (MIS-C) in a pediatric patient.

A 15-year-old biracial female without significant past medical history tested positive for COVID-19 and two weeks later presented with respiratory symptoms and other systemic signs. She was admitted for further evaluation and was found to have elevated inflammatory markers, EKG (electrocardiogram) abnormalities, and lab evidence of organ damage. The patient was diagnosed with MIS-C, and treatment was initiated with eventual discharge. One year after this initial visit, the patient returned to the hospital due to weight loss, difficulty breathing, polyuria, polydipsia, nausea, vomiting, and fatigue. A steroid course for MIS-C treatment had been completed three months prior. Exam and lab results confirmed diabetic ketoacidosis (DKA), and the patient was diagnosed with new-onset type 1 diabetes. Further testing determined that she was glutamic acid decarboxylase 65 (GAD-65) positive. DKA was managed in the hospital, and the patient was subsequently discharged with an insulin regimen and endocrine follow-up. A couple of months later, the patient returned to the emergency department (ED) due to two weeks of dyspnea on exertion and dizziness. Since her previous admission for DKA, the patient had contracted COVID-19 again and recovered from her respiratory symptoms. Physical exam and labs were grossly unremarkable; however, the patient had EKG abnormalities and an episode of severe bradycardia, prompting hospitalization. Thyroid workup revealed thyrotoxicosis due to Graves' disease. Due to intermittent hypotension, adrenal labs were obtained. She was found to have adrenal insufficiency as well, with a positive 21-hydroxylase antibody. Throughout these hospitalizations, the patient suffered from skin and hair changes as well, ultimately requiring dermatological intervention.

## Introduction

Since SARS-CoV-2 first appeared in Wuhan, China, in late 2019, millions of people around the world have contracted the virus and have faced complications from the infection. A feared complication of SARS-CoV-2 infection is the development of multisystem inflammatory syndrome (MIS); for children, this is termed multisystem inflammatory syndrome in children (MIS-C). MIS-C is characterized by at least two signs of multisystem involvement, fever, and elevation of inflammatory markers with confirmation of recent COVID infection [1]. Overall, rates of MIS-C diagnosis remain relatively low, with less than 1% of pediatric patients meeting the criteria [2]. Of the children who are affected, African Americans and Latino children represent the highest proportion [2]. However, a phenomenon that has been increasingly identified after COVID infection is the development of certain autoimmune conditions. There is a rising incidence of new-onset type 1 diabetes after COVID infection, specifically within the pediatric population. The COVID-19 pandemic has been associated with an increasing rate of new diagnoses of type 1 diabetes, from less than 20% in 2019 to over 30% in 2020 worldwide [3]. Along with a higher incidence of cases, the percentage of children presenting in diabetic ketoacidosis (DKA) or severe DKA also increased in 2020 compared to previous years [3]. 

Although we are now discovering relationships between COVID-19 and certain autoimmune conditions like diabetes, the concept of stress-induced autoimmunity is not new. Stress-induced autoimmunity refers to an environmental factor that causes molecular changes in a genetically-predisposed individual, which typically results in phenotypic manifestations of a disease state [4]. Viruses represent a major category of environmental triggers for autoimmune disease, with multiple genetic and molecular mechanisms proposed [5]. Along with type 1 diabetes, studies have noticed associations between recent COVID-19 infection and other autoimmune conditions, such as Graves' disease, immune thrombocytopenic purpura (ITP), and systemic lupus erythematosus (SLE) [6]. However, the development of multiple endocrine disorders, also known as polyendocrine syndrome, after COVID-19 infection has not been a well-established association. Upon literature review, there have been studies examining the impact of COVID-19 in patients with existing polyendocrine syndrome, specifically those with autoimmune polyglandular syndrome (APS)-1, and their risk for severe viral-associated pneumonia [7-9]. Studies examining polyendocrine syndrome that results from COVID-19 infection cannot be found for the adult population. To the best of our current knowledge, only one other case report exists that associates COVID-19 infection with polyendocrine syndrome in a pediatric patient. In that case, however, the pediatric patient developed adrenal insufficiency followed by hypothyroidism shortly after contracting COVID-19 [10]. For our case, we present a pediatric patient who developed type 1 diabetes, Addison's disease, and Graves' disease after presenting with MIS-C from COVID-19 infection. 

## Case presentation

COVID-19

A 15-year-old female with no significant past medical history who tested positive (in fall 2020) for COVID-19 two weeks prior presented to the emergency department with a pruritic rash, diffuse body swelling, fever of 104F, slight tachycardia (with normotensive blood pressure), diarrhea, decreased urine output, sore throat, and mouth sores on the inside of her lower lip. She had recently been on penicillin due to concern for dental hardware infection. A physical exam in the ED showed a young female in moderate distress with diffuse swelling, especially of the face, generalized erythematous maculopapular rash, ulcerations on the inside of her lower lip and posterior portion of the tongue, erythematous posterior pharynx, bilateral cervical lymphadenopathy, and erythematous edematous toes. Differential diagnoses included MIS-C versus penicillin allergy versus serum sickness versus drug fever/rash. The patient was admitted for further workup. 

MIS-C

**Figure 1:**
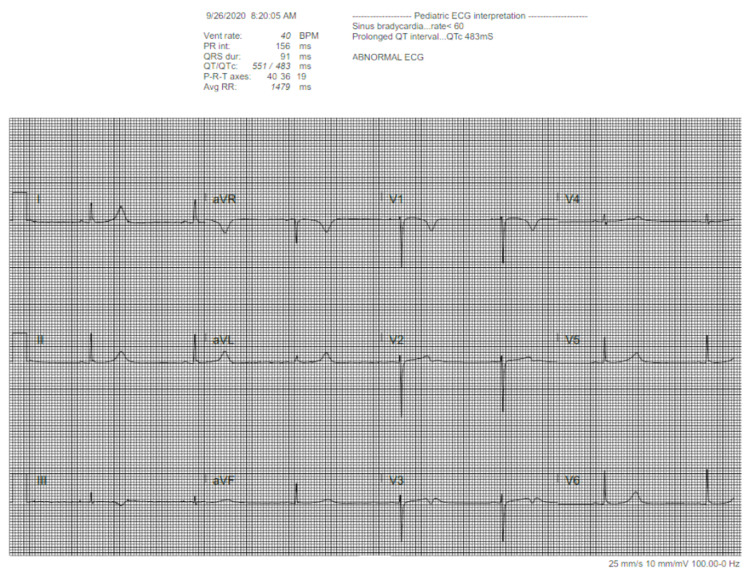
Prolonged QT interval found during MIS-C admission MIS-C - multisystem inflammatory syndrome in children

EKG showed prolonged QT interval (Figure 1), and echocardiogram (ECHO) revealed echogenicity in the left anterior descending (LAD), left coronary, and right coronary arteries, consistent with inflammation. Her labs were remarkable for leukocytosis, markedly elevated D-dimer, elevated C-reactive protein (CRP), low fibrinogen, prolonged PT, transaminitis, elevated lactate dehydrogenase (LDH), and positive severe acute respiratory syndrome (SARS) Ab IgG. Workup was consistent with MIS-C. Rheumatology recommendations included treatment with intravenous immunoglobulin (IVIG), methylprednisolone, and low-dose aspirin. The patient was also started on medications to treat the pruritis associated with her rash. ECHO had normalized by discharge. Her rash had improved; however, she still had scattered petechiae on her palms and intermittent pruritis that typically worsened at night. WBC count, D-dimer, and liver enzymes had all decreased but remained elevated from normal. She was discharged one week later with oral prednisone, and a Holter monitor to follow up on her prolonged QT found on initial presentation. She had a follow-up scheduled with cardiology and rheumatology outpatient. 

Type 1 diabetes

About a year later, the patient presented to the ED with one week of cough, congestion, and intermittent wheezing. These symptoms were accompanied by vomiting, increased thirst and urination, fatigue, and difficulty breathing. She had a 10-pound weight loss within a month. She had been off steroids for three months. She had been seen by her primary care provider the previous day, who reported a glucose of 371. Exam at the ED was significant for tachycardia, Kussmaul breathing, and diffuse abdominal tenderness to palpation. Lab results were significant for glucose 411, critical value of ketones in the urine, and pH of 7.1. The patient was directly admitted to the pediatric intensive care unit (PICU) for the management of diabetic ketoacidosis. She was diagnosed with new-onset Type 1 diabetes; she was determined to be GAD-65 positive (Table 1). Presenting symptoms were resolved by the day of discharge. Her insulin regimen on discharge consisted of 22 units of Lantus daily and a 1:10 insulin-to-carbohydrate ratio for meals with a sliding scale of 1 unit for every 50 mg/dL over 150 mg/dL. The patient was followed by endocrine and cardiology outpatient after this admission. Cardiology recommended yearly EKG, given a history of prolonged QT. 

**Table 1:** Diabetes labs GAD-65 - glutamic acid decarboxylase 65

Diabetes labs​	Value	Reference range
HbA1c	10.3% (high)	<5.7%
GAD-65	42.6 nmol/L (high)​	<0.02

Graves' disease and Addison's disease 

**Figure 2:**
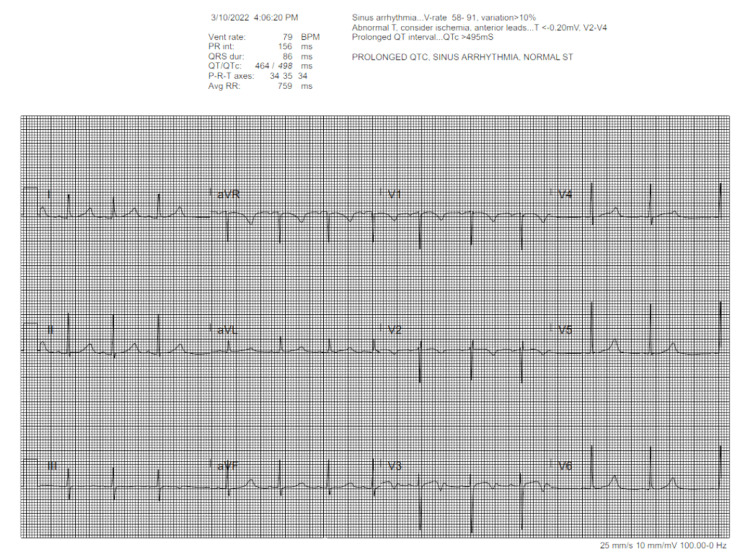
Prolonged QT with T wave inversions

Two months after admission for DKA, the patient presented to ED with dyspnea on exertion and dizziness for toe weeks. Her symptoms had become so severe that she could not shower standing up. She denied having any syncopal episodes and stated that her blood sugars had been well-controlled. The most recent A1c approximately two months prior was 6.9. In the ED, the patient had systolic blood pressure in the upper 90s with diastolic in the 70s, but the physical exam was overall unremarkable. CBC, CMP, CRP, ESR, lactic acid, troponin, BNP, and ABG were within normal limits. CXR was obtained that showed no focal consolidations. EKG demonstrated prolonged QTc, sinus arrhythmia, and T-wave inversions in V1, V2, and V3 (Figure 2). Due to abnormal EKG, cardiology was consulted and recommended giving a fluid bolus. While awaiting bolus, the patient experienced a few seconds of bradycardia down to the 20s-30s, which prompted hospital admission with telemetry. The patient reported that she had contracted COVID for a second time after her admission for DKA. With this course, she had respiratory symptoms lasting less than two weeks, followed by generalized abdominal pain and nausea. Given her lack of fever or elevated inflammatory markers, concern for recurrent MIS-C was low. Her variable heart rate, history of autoimmune conditions, and palpable thyroid on exam prompted evaluation for underlying thyroid disorder and inpatient endocrine consultation. Thyroid labs showed an almost undetectable thyroid stimulating hormone (TSH) and a high free-T4 at 4.77 ng/dL (Table 2). Combined with her symptomatology, her labs suggested thyrotoxicosis. Therefore, further labs were obtained. Her T3 was noted to be elevated, and thyroid stimulating immunoglobulin (TSI), thyroid peroxidase (TPO) antibody, and thyroglobulin (TG) antibody all came back positive (Table 2). She was then started on Methimazole and Propranolol. Due to occasional hypotension and complaint of dizziness as well as nausea, she was also screened for adrenal insufficiency with a morning cortisol level. Morning cortisol was found to be low at 2.56 mcg/dL (Table 3). The low cortisol level triggered additional screening with a cortisol stimulation test, which only raised cortisol levels to 13.79 (Table 3). She was given a one-time stress dose of hydrocortisone with improvement of symptoms. Further testing showed positive 21-hydroxylase antibody (Table 3). 

**Table 2:** Thyroid labs TSH - thyroid-stimulating hormone,  TSIg - thyroid-stimulating immunoglobulins, TPO - thyroid peroxidase

Thyroid labs​	Value	Reference range
TSH	< 0.01 mcIU/mL (low)	0.4 - 4.7
TSIg	3.7 (high)	<1.3
Free T4	4.77 ng/dL (high)	0.58 - 1.76
TPO Ab	102.7 IU/mL (high)	<9.0
Free T3	17.61 pg/mL (high)	2.3 - 4.2
Thyroglobulin level	636 ng/mL (high)	<33
T3	5.54 ng/mL (high)	0.6 - 1.81
Thyroglobulin Ab	<1.8​ IU/mL	<1.8

**Table 3:** Adrenal labs 21-OH - 21-hydroxylase, ACTH - adrenocorticotropic hormone

Adrenal labs​	Value	Reference range
AM cortisol	2.56 mcg/dL (low)	3.09 - 22.4
Stim cortisol	13.79 mcg/dL​	3.09 - 22.4
21-OH Ab​	Positive	
ACTH	13 pg/mL	7.2 - 63

Skin and hair effects 

Throughout the course of these multiple hospitalizations, the patient suffered from dramatic skin changes. These included skin hypo- and hyper-pigmentation, intense pruritis, roughened skin texture, and hair loss, specifically in the eyebrows and scalp. She required dermatological intervention with medications such as hydroxyzine, triamcinolone, and dupilumab for several months but has now improved with only mild dyspigmentation remaining. 

## Discussion

COVID-19 and MIS-C

In this report, we present a case of polyendocrine syndrome developing after multisystem inflammatory syndrome (MIS) in a pediatric patient with no known prior health conditions. At the time of her presentation, adolescents aged 14-17 comprised 16.3% of the almost 3 million laboratory-confirmed COVID-19 cases in people aged 0-24 [2]. By the end of 2020, there were slightly less than 20 million confirmed cases of COVID-19 in the United States [11], meaning that children and adolescents represented approximately 15% of positive cases. Therefore, of this 15%, only about 2.45% were those aged 14-17. Development of MIS-C after a COVID-19 infection is an even rarer occurrence, with current estimates suggesting <1% of children with confirmed SARS-CoV-2 infection who meet the criteria for MIS-C. The Centers for Disease Control (CDC) and the World Health Organization (WHO) use slightly different criteria to define MIS-C; while the CDC requires symptoms severe enough to require hospitalization, the WHO does not. Both definitions are similar in that they require proof of recent COVID infection, at least two signs of multisystem involvement, fever, and elevated inflammatory markers without another plausible diagnosis [1]. Of the children who develop MIS-C, those who identify as Black or Hispanic/Latino comprise a larger portion of cases than other racial/ethnic groups. Our patient is of mixed descent, having both a Caucasian parent and an African American parent. Data on the development of MIS-C in children of mixed descent is limited. 

Virally-induced autoimmunity

At present, only one other study exists that establishes a link between development of autoimmune polyendocrine syndrome and recent COVID infection [4]. However, the concept of virally-induced autoimmunity is a well-recognized phenomenon. The hypothesized mechanisms by which viruses can cause immune system dysregulation include molecular mimicry, bystander activation, and epitope spreading, among others. An example of such a phenomenon is the onset of type 1 diabetes after enteric infection with the Coxsackie B virus (CVB). Research suggests that molecular mimicry between the CVB protein of the virus and the GAD65 enzyme in pancreatic beta cells leads to functional impairment of beta cells, ultimately resulting in inflammation that produces autoantigens and subsequent diabetes development in hosts already predisposed. These host factors can include factors such as genetic predisposition and time of infection. Rheumatoid arthritis, multiple sclerosis, and systemic lupus erythematous represent other diseases for which viral triggers have been examined [5]. 

COVID-19 and endocrine disorders

COVID-19 viral mRNA can be detected in blood, stool, and urine. The virus can interact with angiotensin-converting enzyme 2 (ACE-2) and transmembrane serine protease 2 (TMPRSS2) in other organs as well [12]. ACE-2 receptors are the main binding site of the virus, particularly for the coronavirus spike protein [12]. ACE is expressed in pancreatic beta cells [12]. The pancreas, hypothalamus, pituitary, thyroid, adrenals, testes, and ovaries express ACE2 receptors [12]. The highest concentration of ACE2 and TMPRSS2 exists in endocrine glands, specifically the testes and thyroid; it is lowest in the hypothalamus [12]. Data is scarce regarding endocrine disorders during the infectious phase and recovery phase [13]. Does hyperinflammation and cytokine storm in infection expedite the onset of type 1 diabetes mellitus (T1DM) in those already genetically susceptible? [14] Available data shows that glucose dysregulation causes new-onset diabetes. Data also shows thyroid dysfunction can occur, namely subacute thyroiditis, as evidenced by low T3 and low TSH. This could be due to multiple possible mechanisms, such as the glucocorticoid effect, direct virus effect on follicular cells, and immune mechanisms. In addition, adrenal dysregulation and impaired spermatogenesis have been observed as well [13]. 

COVID-19 and Diabetes

Those with diabetes who are hospitalized have the highest risk of comorbidities with SARS-COV-2; it may worsen glucose homeostasis.  Glycemic control plays a role in regulating inflammatory response, preserving tissue integrity and physiological function. Corticosteroids and antivirals used in the treatment of SARS-COV-2 might further aggravate hyperglycemia via insulin resistance or lipodystrophy. Decreased exercise capacity, cachexia, and muscle weakness may diminish insulin sensitivity, especially in acute respiratory distress syndrome (ARDS) and sepsis patients. Rhabdomyolysis has been reported, which can contribute to glucose dysregulation as well. Recent clinical evidence has suggested that SARS-COV-2 in DKA and HHS patients has led to higher-than-usual doses of insulin to control blood glucose. Damage of pancreatic islets by SARS-COV-2 and loss of insulin secretion can be explained by a number of possible mechanisms. The release of chemokines and cytokines might affect pancreatic cells, impairing their ability to sense glucose concentrations and leading to inappropriate amounts of insulin. High expression of ACE2 in beta cells and the beta cells' permissiveness to SARS-COV-2 can induce inflammatory cytokine release, beta cell apoptosis, and decreased insulin secretion. SARS-COV-2 infects and replicates human islets, leading to morphological, transcriptional, and functional changes. This reduces insulin secretory granules and impairs glucose-dependent insulin secretion. [13] 

COVID-19 and Thyroid Function

Thyroid function in 50 patients with SARS-COV-2 was investigated three months after diagnosis. Sixty-four percent had abnormal thyroid function.  The degree of decrease of thyroid-stimulating hormone (TSH) and T3 was positively correlated with the severity of the disease. The pathogenesis is not completely understood. The SARS-COV-2 genome has been detected on thyroid samples. ACE2 is highly expressed in thyroid tissue, and therefore, direct damage to the thyroid gland is possible. There could be an underlying euthyroid sick syndrome caused by critical illness; this is a homeostatic mechanism to recover from severe illness. Drugs used for managing SARS-COV-2 can affect thyroid function. Glucocorticoids could affect serum TSH levels by inhibiting thyrotropin-releasing hormone (TRH) secretion or by suppressing TSH release.  Heparin causes displacement of total T4 from thyroid-binding globulin, leading to higher measured free-T4 [13]. There are three case reports of subacute thyroiditis, four cases of autoimmune hyperthyroidism, and Graves' disease after SARS-COV-2 infection. Diagnosis is usually within 1-2 months after clinical onset of SARS-COV-2. Thiamazole and propranolol were used for treatment, leading to the improvement of symptoms [15]. 

COVID-19 and Adrenal Function

Adrenal glands play a crucial role in the immune response as they secrete cortisol and catecholamines. Those with known adrenal insufficiency and Cushing's syndrome present with higher susceptibility to infections. A few clinical cases have been reported of adrenal hemorrhage as a complication. Autopsy studies show adrenal microinfarction or adrenal lesions [13].  The European Society of Endocrinology (ESE) stated there is no evidence that patients with adrenal insufficiency are at increased risk of contracting COVID-19. In addition, there is no reported data on the outcomes of infection in those with adrenal insufficiency [7]. A 32-year-old woman in Italy with autoimmune polyglandular syndrome type 1 (APS-1) contracted SARS-COV-2; her mutation of the autoimmune regulator (AIRE) gene led to primary adrenal insufficiency (PAI), hypoparathyroidism, hypogonadism, ectodermal dystrophy, candidiasis, pernicious anemia, and gastrointestinal dysfunction [7]. There is understandably impaired natural immunity function in PAI; there is defective action of neutrophils and natural killer cells. Exogenous glucocorticoids may play a role in modulating the immune system. Patients with APS-1 have primary immunodeficiency, explaining the T-cell deficiency-related chronic mucocutaneous candidiasis [7]. There is evidence that suggests that those with Addison's disease have a higher risk of infections of the lower respiratory, urinary, and gastrointestinal tracts. Also noteworthy, compared to those untreated for congenital adrenal hyperplasia (CAH), those who were treated had an increased risk of infection; non-physiological glucocorticoid replacement may be a risk factor for developing infection [7]. 

Autoimmune polyendocrine dilemma 

The coexistence of Graves' disease and adrenal insufficiency (Addison's disease) can make for an interesting clinical picture, as Graves' disease typically results in hypertension and tachycardia, whereas adrenal insufficiency manifests as hypotension and bradycardia. However, in our patient, the predominant symptomatology aligned with that of Graves' disease, given her elevated blood pressure and heart rate. The coexistence of Addison's disease and type 1 diabetes is unique as well, given that Addison's disease tends to cause hypoglycemia, whereas diabetes causes hyperglycemia. However, this patient did not report hypoglycemic episodes. 

Autoimmune conditions associated with T1DM 

Even outside of the context of viral induction, type 1 diabetes has been associated with other autoimmune conditions. These include gastrointestinal disorders such as pernicious anemia and celiac diseases, thyroid disorders, adrenal insufficiency, and vitiligo. Common autoimmune diseases tend to occur together. Patients with a combination of type 1 diabetes and autoimmune diseases were older and were more likely female compared to patients with only type 1 diabetes. Gastrointestinal autoimmune diseases, such as type A gastritis and celiac disease, are more common in patients with combined disease. Hashimoto thyroiditis is the most frequent autoimmune thyroid disease. Glutamic acid decarboxylase antibody (GAD-Ab) is more commonly found in those diagnosed after adolescence and is usually present at the clinical presentation of T1DM. GAD-Ab positivity was more frequent in patients with combined type 1 diabetes and autoimmune disease. All with combined type 1 diabetes and autoimmune diseases with positive thyroid Ab had thyroid dysfunction. About a third of T1DM patients will develop thyroid antibodies and thyroid dysfunction [16]. 

Autoimmune polyendocrine syndrome

After MIS-C, our patient sequentially developed type 1 diabetes mellitus, Graves' disease, and, finally, adrenal insufficiency. This combination of multiple endocrinopathies that arise from immune system dysregulation is known as autoimmune polyendocrine syndrome. Autoimmune polyendocrine syndrome (APS) is a rare condition, with the prevalence of APS type 1 at 1 in 100,000 and that of APS types 2-4 at 1 in 20,000. APS-1 is also known as APECED syndrome and is more commonly diagnosed in childhood. This syndrome is autosomal recessive in inheritance and is caused by impairment of the AIRE gene, which leads to the development of autoreactive lymphocytes. Endocrine manifestations of APS-1 include adrenal failure, hypoparathyroidism, and hypogonadism. APS-2 is multifactorial in its inheritance; however, certain human leukocyte antigen (HLA) class 1 and class 2 alleles have been associated with this syndrome, as well as single nucleotide polymorphisms in some genes. APS-2 is more common than the recessive APS-1 and is associated with a combination of type 1 diabetes, adrenal insufficiency, and autoimmune thyroiditis. One must have at least two of these three endocrinopathies to be considered for APS-2. While APS-1 tends to present in younger children, APS-2 presents at its earliest in adolescence and has a higher prevalence in females [17].

COVID and APS-1

Patients with APS-1 have autoantibodies against Th17 cytokines IFN-α and IFN-ω (type I IFNs). The role of autoantibodies against type I IFNs for infectious diseases has been suspected; patients with APS-1 have developed severe infections from COVID-19 [11]. Patients with APS-1 can have autoantibodies at titers sufficient for functional neutralization of type I IFNs; type I IFN-mediated inhibition of COVID-19 replication is abolished by autoantibodies in patients' plasma in vitro. Reportedly, in four patients with APS-1, also infected with COVID-19, symptoms were mild despite high titers of neutralizing autoantibodies against type I IFNs. This contrast was reported in three patients with APS-1 who had severe symptoms of COVID-19. It is possible these autoantibodies do not fully neutralize either type I IFN in vivo. All patients with APS-1 who contract COVID-19 should be followed closely [11]. 

COVID and APS-2

There is very little data regarding APS-2 manifesting with COVID. Currently, there is only one other case report describing APS-2 manifesting after COVID-19 infection [10]. In that case, an adolescent patient developed primary adrenal insufficiency and autoimmune hypothyroidism just weeks after presumed COVID-19 infection [10]. There also is a case report of adrenal crisis in APS-2 patients due to COVID [18], but other studies examining the occurrence of APS-2 with COVID-19 are lacking. 

APS-2 Classification

We believe our patient most fits an APS-2 picture. APS-1 classically is a pediatric syndrome but includes hypoparathyroidism, adrenal insufficiency, and mucocutaneous candidiasis. Clinically it appears by age 15. APS-2, in contrast, includes a combination of adrenal insufficiency, thyroid dysfunction, and T1DM. Given the difference in clinical manifestation, our patient most closely fits an APS-2 picture. Both manifest with nonendocrine organ dysfunction, including dermatologic and gastrointestinal manifestations [19]. APS-2 conditions may occur in any order and at any age but typically occurs in midlife with adrenal insufficiency as the initial abnormality. Common signs and symptoms include fatigue and skin changes, sometimes patches of vitiligo as well; hypotension, hypoglycemia, and hyponatremia are late manifestations. Our workup for Addison's disease was triggered by skin changes we observed. This patient had signs of alopecia, including thinning of her eyebrows; alopecia occurs in 25% of patients [20]. 

Limitation

We acknowledge that a limitation of our case report is that we see a causative link in our patient between MIS-C and APS2 when in reality, it could be that these findings are coincidental and unrelated. As more cases appear, we may have a greater understanding of that possible link. 

## Conclusions

Our patient is a unique case of poly-endocrine syndrome post-COVID with a triad of T1DM, Graves' disease, and Addison's disease. There is only one other published case of APS-2 post-COVID in a pediatric patient. Our case differs in that our patient has not only thyroid dysfunction and adrenal insufficiency but also T1DM. This case report further explores the connection between APS-2 and COVID-19 infection in a pediatric patient and represents an area of further study for COVID-19-related sequela. 
